# Deciphering the impact of contaminating microbiota in DNA extraction reagents on metagenomic next-generation sequencing workflows

**DOI:** 10.1128/spectrum.03119-24

**Published:** 2025-08-20

**Authors:** Zi-Lun Lai, Yang-Di Su, Hsiu-Hsien Lin, Szu-Yun Wang, Yu‑Chao Lin, Shinn‑Jye Liang, Wei-Cheng Chen, Po-Ren Hsueh

**Affiliations:** 1 Department of Laboratory Medicine, China Medical University Hospital, China Medical University 38020, Taichung, Taiwan; 2 Division of Pulmonary and Critical Care, Department of Internal Medicine, China Medical University Hospital 38020, Taichung, Taiwan; 3 Graduate Institute of Biomedical Sciences and School of Medicine, College of Medicine, China Medical University 38019, Taichung, Taiwan; 4 Department of Education, China Medical University Hospital, China Medical University 38019, Taichung, Taiwan; 5 Department of Infectious Diseases, Department of Internal Medicine, China Medical University Hospital, China Medical University 38020, Taichung, Taiwan; 6 PhD Program for Aging, School of Medicine, China Medical University 38019, Taichung, Taiwan; Ann & Robert H. Lurie Children's Hospital of Chicago, Chicago, Illinois, USA

**Keywords:** mNGS, contamination, DNA extraction reagents, background microbiota, lot variability, microbiome, bioinformatics, clinical diagnostics

## Abstract

The widespread adoption of metagenomic next-generation sequencing has revolutionized microbial detection, yet contaminating DNA in laboratory reagents poses significant challenges for result interpretation. This study investigated microbial contamination profiles across four commercial DNA extraction reagent brands (M, Q, R, and Z) and assessed batch-to-batch variability. Extraction blanks were generated using molecular-grade water or ZymoBIOMICS Spike-in Control I as input materials. Analysis revealed distinct background microbiota profiles between brands, with some containing common pathogenic species that could affect clinical interpretation. Notably, background contamination patterns varied significantly between different lots of the same brand, highlighting the need for lot-specific microbiota profiling. Site-specific environmental contaminants were identified through analysis of 30 control samples from a single study site. Additionally, comparison of blood samples from healthy individuals with control samples suggested no evidence of a consistent blood microbiome, suggesting that “extraction blanks” may serve as negative controls in clinical metagenomic testing of sterile liquid biopsy samples. These findings emphasize the importance of including negative controls in every run and underscore the need for manufacturers to provide comprehensive background microbiota data for each reagent lot to optimize clinical interpretation and minimize false-positive results.

**IMPORTANCE:**

Metagenomic next-generation sequencing (mNGS) has revolutionized pathogen detection and microbiome studies, but contamination from DNA extraction reagents remains a critical challenge. This study highlights the significant variability in background microbiota profiles across reagent brands and manufacturing lots, emphasizing the need for manufacturers to provide detailed contamination profiles. Our findings underscore the importance of implementing extraction blanks as standard controls and incorporating bioinformatics tools to account for background noise. These measures are essential to enhance the reliability of mNGS results and prevent diagnostic errors, particularly in clinical settings where contamination could mask or mimic pathogen signals. Additionally, our confirmation that healthy blood lacks a consistent microbiome helps streamline control selection in clinical testing protocols, potentially reducing costs and complexity in clinical mNGS workflows.

## INTRODUCTION

The advent of next-generation sequencing (NGS) has led to an explosion of shotgun metagenomic NGS (mNGS) studies in the past decade (1, 2). Metagenomics is defined as the direct analysis of whole microbial communities based on nucleic acids (3) extracted from clinical samples, enabling an agnostic approach for detecting unknown and non-culturable microorganisms. However, shotgun mNGS also detects nucleic acids from contaminants, which can confound the interpretation of microbiome data (4, 5) and lead to erroneous disease diagnoses (6, 7). Such contamination effects are common, as several studies have identified contaminant microbial DNA in laboratory reagents, surfaces, and environments (6, 8).

Contaminants can be classified as either external or internal. External contaminants originate from outside the samples and may include DNA from patient or investigator skin, clinical and laboratory equipment, collection tubes, contaminated laboratory surfaces or air, extraction kits, library preparation reagents, and even molecular biology-grade water (9). While mNGS reagent manufacturers do not guarantee the absence of contaminating DNA in their products, even reagents/kits sold as sterile may contain DNA (10). External contaminations have unique profiles specific to particular reagents—referred to as “kitomes”—that vary across different extraction kits, batches of the same reagent, laboratories, and time periods (11). Extraction kits appear to be the major source of external noise in microbiome studies (6, 12). More contamination has been reported for manual compared to automated extraction (13), and RNA sequencing is more susceptible to contamination than DNA sequencing (14).

To address external contamination, Benjamin Callahan and collaborators developed Decontam, a software package designed for identifying and removing contaminant sequences computationally in mNGS data (15). Decontam makes use of a statistical classification procedure to identify contaminants in mNGS data based on a pattern whereby contaminants appear at higher frequencies in low-concentration samples and are found in negative controls. Other similar tools designed to identify and remove contamination from microbial community samples at the biocomputational level include microDecon (16) and SourceTracker (17). One prerequisite is the availability of sensitive wet-lab approaches that can precisely detect potential contamination, enabling subsequent decontamination through biocomputational analysis.

**Fig 1:**
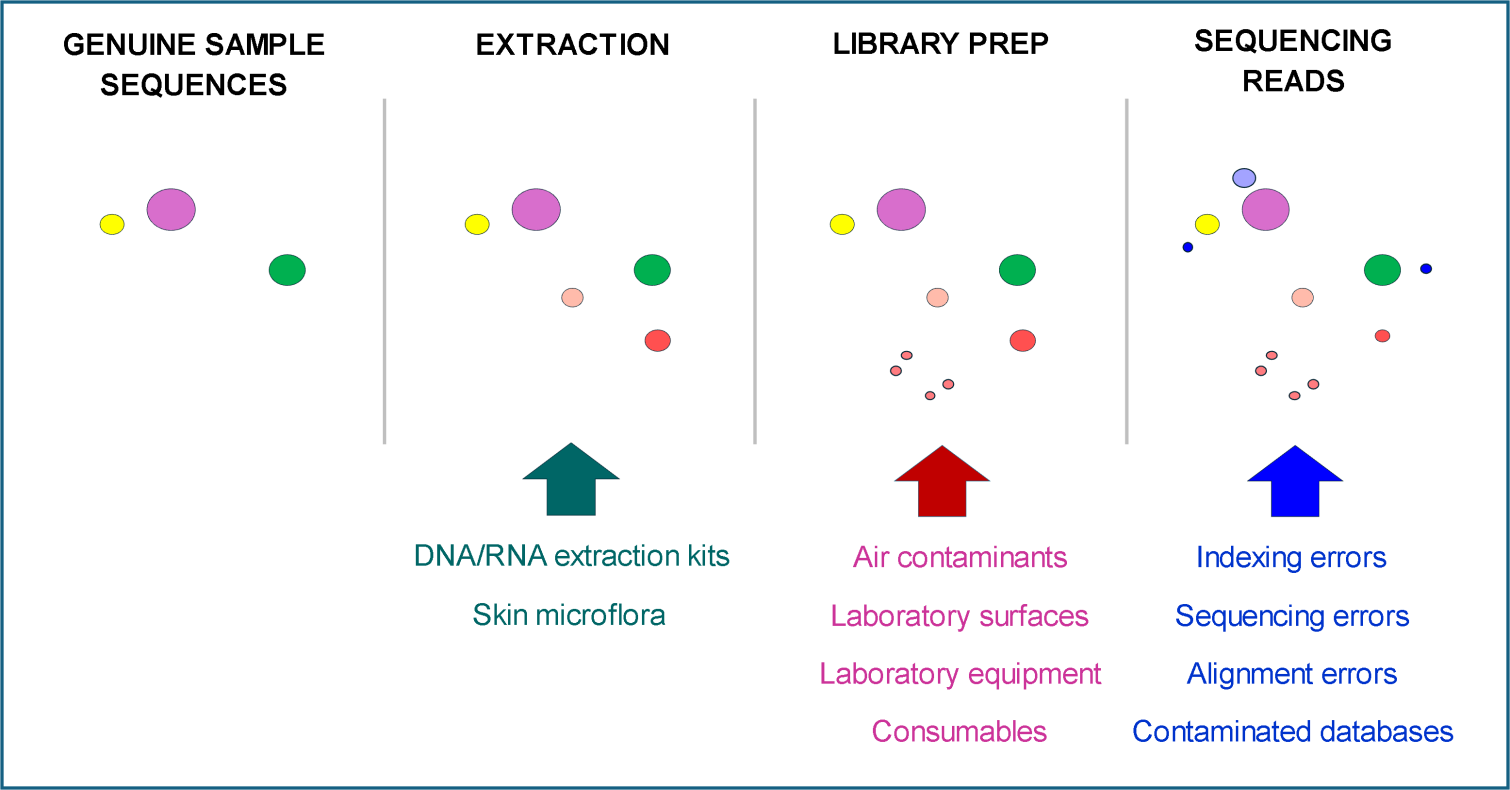
Sources of contamination in the mNGS workflow. Schematic representation of contaminants introduced during the mNGS workflow, from sample collection through bioinformatics analysis that may interfere with the identification of genuine pathogen-derived microbial DNA sequences. External contamination sources are indicated in red text, while internal contamination sources are shown in blue text. The workflow demonstrates the multiple points where contamination can be introduced, affecting the accuracy of pathogen detection.

Internal contamination may arise from sample mix-up or well-to-well contamination at various workflow steps. Run-to-run contamination has been observed with MiSeq Sequencing System (Illumina, USA) (8). Another type of internal contamination, known as “index hopping” or “index switching,” leads to incorrect sample assignment of sequencing reads in multiplexed sequencing runs (18). Internal contamination may also result from erroneous bioinformatic read classification (19, 20). The possible sources of external and internal contamination are summarized in Fig. 1.

For infectious disease applications of mNGS, recent years have seen considerable interest in the potential existence of a microbiome in healthy individuals' blood, with some studies suggesting the presence of multiple circulating microbial species in healthy human blood (21). Tan et al. (21) analyzed microbial DNA in blood samples from 9,770 healthy individuals and identified 117 microbial species distinct from pathogens typically detected in hospital blood cultures. No species were detected in 84% of individuals, while the remainder had a median of one species, and less than 5% of individuals shared the same species. These results did not support the existence of a consistent core microbiome endogenous to human blood, instead suggesting transient and sporadic translocation of commensal microbes from other body sites into the bloodstream.

This study aims to profile the background microbiota in common DNA extraction reagents used for mNGS and to understand the reproducibility of this background between manufacturing lots and replicate runs. Notably, the intended application of the mNGS workflow in this study is for pathogen identification in a clinical setting (22). As such, we analyzed blood samples from healthy controls to investigate whether these samples might have a “microbiota background” that could indicate the presence of a blood microbiome.

## MATERIALS AND METHODS

### Sample collection

Peripheral venous blood (20 mL) was collected from healthy adults and patient volunteers under informed consent, as approved by the Institutional Review Board of the China Medical University Hospital (CMUH111-REC1-074 and CMUH112-REC1-207). Samples were processed immediately, and DNA was extracted from 10 mL of each whole blood sample using the protocols described below.

### DNA extraction

Extraction blanks were generated from four brands of DNA extraction reagents: Micronbrane (M) and other brands Q, R, and Z. Extraction protocols were executed using either molecular-grade (DNA-free) water (indicated as MBG) (Product number W4502-1L; Sigma-Aldrich) or ZymoBIOMICS Spike-in Control I (D6320, ZYMO RESEARCH, USA) (indicated as SICP) as input. D6320 consists of equal cell numbers of two bacterial strains, *Imtechella halotolerans* and *Allobacillus halotolerans*. When spiked into a sample, this product serves as an *in situ* positive control for extraction and sequencing. The MBG product was 0.1 µm filtered and was suitable for molecular biology applications. This product has been analyzed for the absence of nucleases and proteases and has undergone bioburden analysis. The experimental design is summarized in Table 1. All tests were conducted in triplicate. For the M brand, DNA extraction was performed using the DEVIN Microbial DNA Enrichment Kit (Micronbrane Medical, Taiwan) according to the manufacturer’s instructions without deviation. For Q, R, and Z brands, the extraction kits used were the QIAamp DNA Microbiome Kit (Qiagen, Germany), MagCore Genomic DNA Whole Blood Kit (RBC Bioscience, Taiwan), and ZymoBIOMICS DNA Miniprep Kit (ZYMO RESEARCH), respectively. These were also performed according to the manufacturer’s instructions without deviation. DNA extractions for each kit/lot were done on separate days.

**TABLE 1:** Summary of experimental design and testing conditions

Brand of extraction kit	Lot	Molecular biology grade (MBG) water input		ZymoBIOMICS spike-in control (SICP) D6320 input	
		Denotation	Triplicates	Denotation	Triplicates
M (Micronbrane)	1	MA-MBG	MEK-01-MBG1-MO1019 MEK-01-MBG2-MO1019 MEK-01-MBG3-MO1019	–^*a*^	–
	2	MB-MBG	MEK-01-MBG1-1109211 MEK-01-MBG2-1109211 MEK-01-MBG3-1109211	–	–
	3	MD-MBG	MEK-01-MBG1 MEK-01-MBG2 MEK-01-MBG3	MEK-01-SICP	MEK-01-SICP1 MEK-01-SICP2 MEK-01-SICP3
Q	1	Q-MBG	Q-MBG1 Q-MBG2 Q-MBG3	Q-SICP	Q-SICP1 Q-SICP2 Q-SICP3
	2	Q-15-MBG	Q-15-MBG1 Q-15-MBG2 Q-15-MBG3	–	–
	3	Q-19-MBG	Q-19-MBG1 Q-19-MBG2 Q-19-MBG3	–	–
R	1	R-MBG	R-MBG1 R-MBG2 R-MBG3	R-SICP	R-SICP1 R-SICP2 R-SICP3
Z	1	Z-MBG	Z-MBG1 Z-MBG2 Z-MBG3	Z-SICP	Z-SICP1 Z-SICP2 Z-SICP3

^*a*^ "–” indicates not available.

### mNGS library preparation and sequencing

All resultant eluates underwent library preparation using Unison Ultralow DNA NGS Library Preparation Kit (Micronbrane Medical). After DNA tagmentation, PCR amplification was performed under the following conditions: (Top lid at 103°C); 68°C, 3 min; 98°C, 3 min; 14 cycles of 98°C, 45 s/62°C, 30 s/68°C, 2 min; 68°C, 1 min; 10°C, ∞. After amplification, the reaction was purified using Sera-Mag Select (Cytiva, USA) and eluted with 11 µL of Elution Buffer. 10 µL of supernatant was transferred to a new DNA LoBind Tube (Eppendorf, Germany) and stored at −20°C. The sequencing libraries were sequenced either single-ended by a MiSeq V3 150 bp kit, by paired-end 150 bp by NovaSeq 6000 in this study. The analysis was run on single-end sequence data to test the feasibility of fast turnaround time in clinical settings.

### Bioinformatics analysis

All sequencing reads were processed to remove adapters and underwent quality trimming at an average *Q* score threshold of 30 (<*Q*30) using fastp (v0.23.2) (23). Raw reads were mapped to a human reference genome (GRCh38) using BWA-MEM (version 0.7.17-r1188). The remaining microbial reads were aligned to the microbial database using Burrows–Wheeler Aligner software (24).

Our custom testing reference database was derived from the NCBI Assembly and Genome databases (25). However, the full RefSeq database is comprehensive but computationally intensive to use. Running analyses against the entire database would require significantly more processing power and time, which would be unrealistic in a clinical setting. Therefore, to improve efficiency, 1,540 microorganisms from 484 unique genera were referenced from pathogen lists of relevant clinical laboratories in China Medical University Hospital and other medical centers in Taiwan. This database is intended as a comprehensive diagnostic reference, inclusive of well-characterized pathogens, emerging or opportunistic species, and environmental species with reported, though rare, clinical associations. DNA viruses and parasites of diagnostic significance are included. In general, one reference genome or the most complete genome available is included for each species to optimize the mapping efficiency. More than one genome is included for certain species when the reference genome is not comprehensive enough to capture reads from other strains of interest. Nevertheless, a robust, clinically ready version database needs to be supported by validation studies and diagnostic relevance. Selection of the reference genomes as well as the complexities introduced by mobile genetic elements needs to be thoroughly tested with clinical samples for clinical accuracy.

BWA was used on the basis of its base-level accuracy and exact read-to-reference alignment, especially in mock community or benchmarking contexts. Metagenomic classifiers like Kraken/Bracken/Kaiju are better for exploratory taxonomic profiling at scale, but less precise at the individual read level.

Principal component analysis (PCA) was performed based on the relative abundance profiles of microbial species in each sample. The abundance data were first normalized (centered and scaled), and Euclidean distance was used to compute sample similarities. The PCA and visualization were conducted using the SRplot online platform (https://www.bioinformatics.com.cn/srplot), a web-based tool for statistical analysis and high-quality scientific plotting. Default settings were applied unless otherwise specified.

## RESULTS

### Comparison across brands M, Q, R, and Z extraction reagents

**Fig 2:**
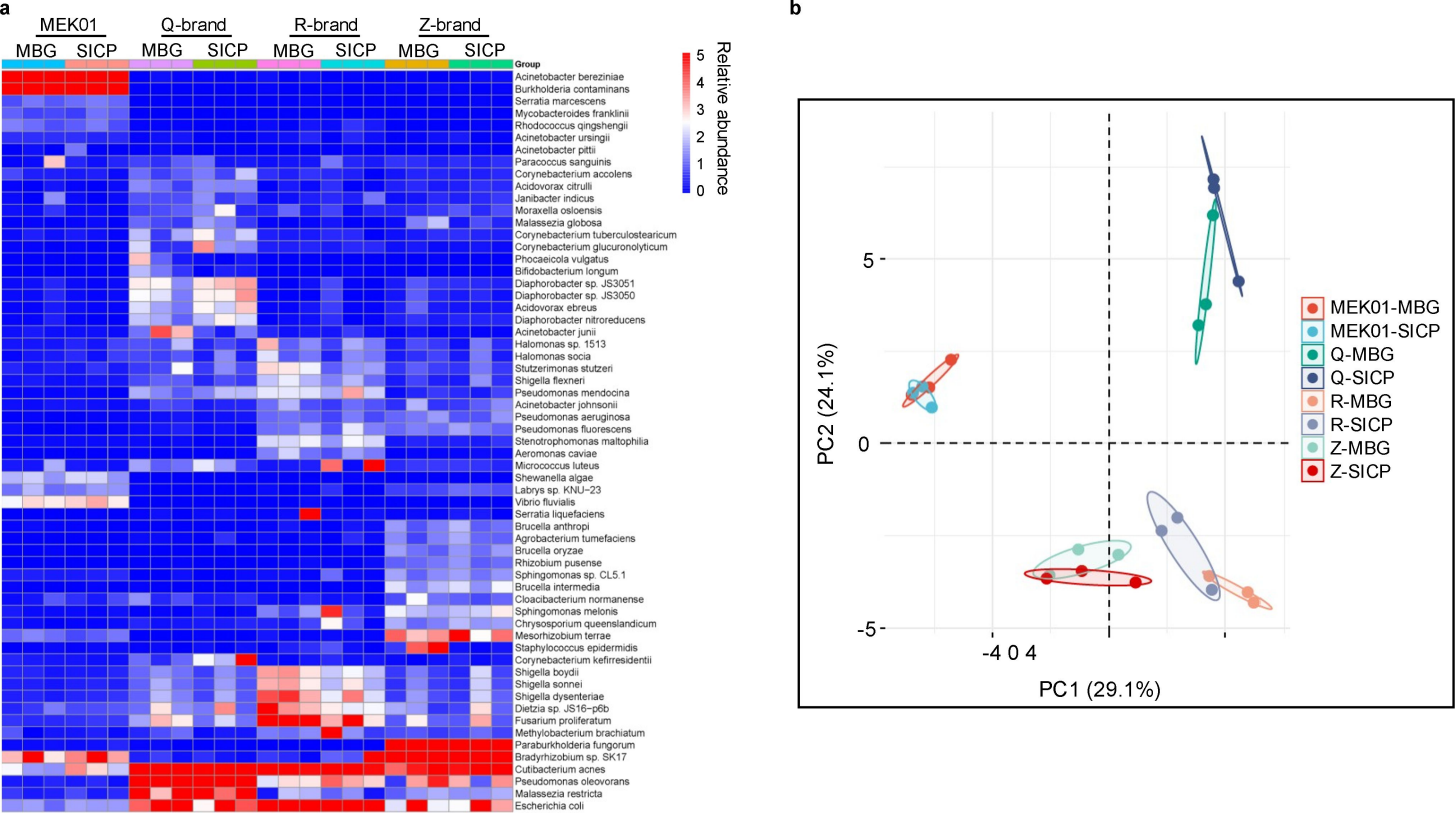
Comparative analysis of background microbiota across different extraction kit brands. Comparison of background microbiota profiles between extraction kit brands M (indicated as MEK01), Q, R, and Z using either molecular-grade (DNA-free) water (MBG) or ZymoBIOMICS Spike-in Control D6320 (SICP) as input material. (a) Heatmap showing the relative abundance of microbial species detected across different brands and input types. (b) Principal component analysis (PCA) demonstrating the clustering patterns of microbial profiles from different brands and input materials.

**Fig 3:**
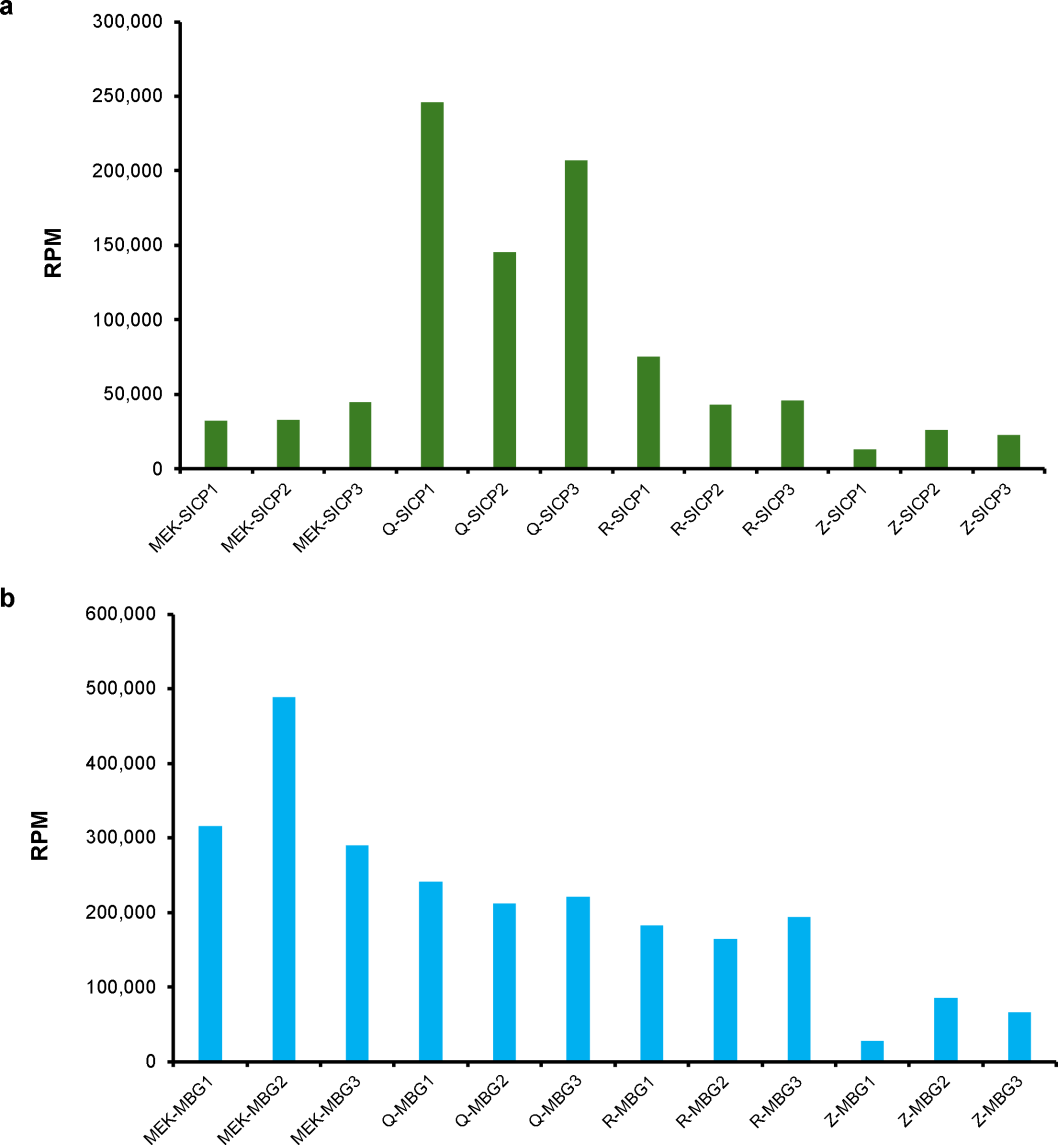
Comparison of normalized microbial sequencing reads across different extraction kits. Sequencing results expressed as reads per million (RPM) for samples processed using extraction kits M, Q, R, and Z. (a) Results from ZymoBIOMICS Spike-in Control D6320 (SICP) samples. (b) Results from molecular-grade (DNA-free) water (MBG) samples. Microbial RPM values represent microbial sequencing reads normalized to 1 million total sample reads, enabling direct comparison of microbial background levels across different extraction kits.

Heatmaps and PCA plots derived from extraction blanks, including MBT and SICP samples from brands M, Q, R, and Z, provide a comparative visualization of microbial profiles across different reagent brands (Fig. 2a and b). These heatmaps and PCA were generated using the microbial reads percentage of detected microbial targets from each sample, enabling clear differentiation of contamination patterns among the brands. Results indicated that background microbiota profiles differed between brands. MBG and SICP showed similar profiles, suggesting that the ZymoBIOMICS Spike-in Control I reference does not contribute additional contaminating bacteria beyond the two intended bacterial strains. Therefore, most background contamination likely originates from the extraction reagents themselves, with spurious microbial detections potentially attributable to random environmental contamination events. The M brand demonstrated the cleanest profile, with *Acinetobacter berezeniae* and *Burkholderia contaminans* as the main contaminants, neither of which are common sepsis pathogens. *Escherichia coli* and *Cutibacterium acnes*, common infection-associated pathogens, were detected in all kits except the M brand. Their presence in the background noise may decrease detection sensitivity and increase false-negative risk. Reads per million (RPM) values represent sequencing reads normalized to 1 million total QC-passed sample reads. This enables direct comparison of background levels across different extraction kits. For SICP samples, the Q-brand exhibited the highest normalized microbial reads (microbial RPM), whereas the M, R, and Z brands demonstrated comparably low RPM values (Fig. 3a). In contrast, this effect was absent in MBG samples, where all brands, except Z, displayed similar RPM values (Fig. 3b). We hypothesize that basal levels of *A. halotolerans* and *I. halotolerans* DNA may modulate PCR amplification during library preparation, such that without spiked bacteria, PCR becomes highly sensitive to background noise. Summaries of the metagenomic output and quality statistics are presented in Tables S1 and S2, which would lend confidence that the sequencing depth was adequate for the analysis performed.

### Comparison between three lots of M and Q

**Fig 4:**
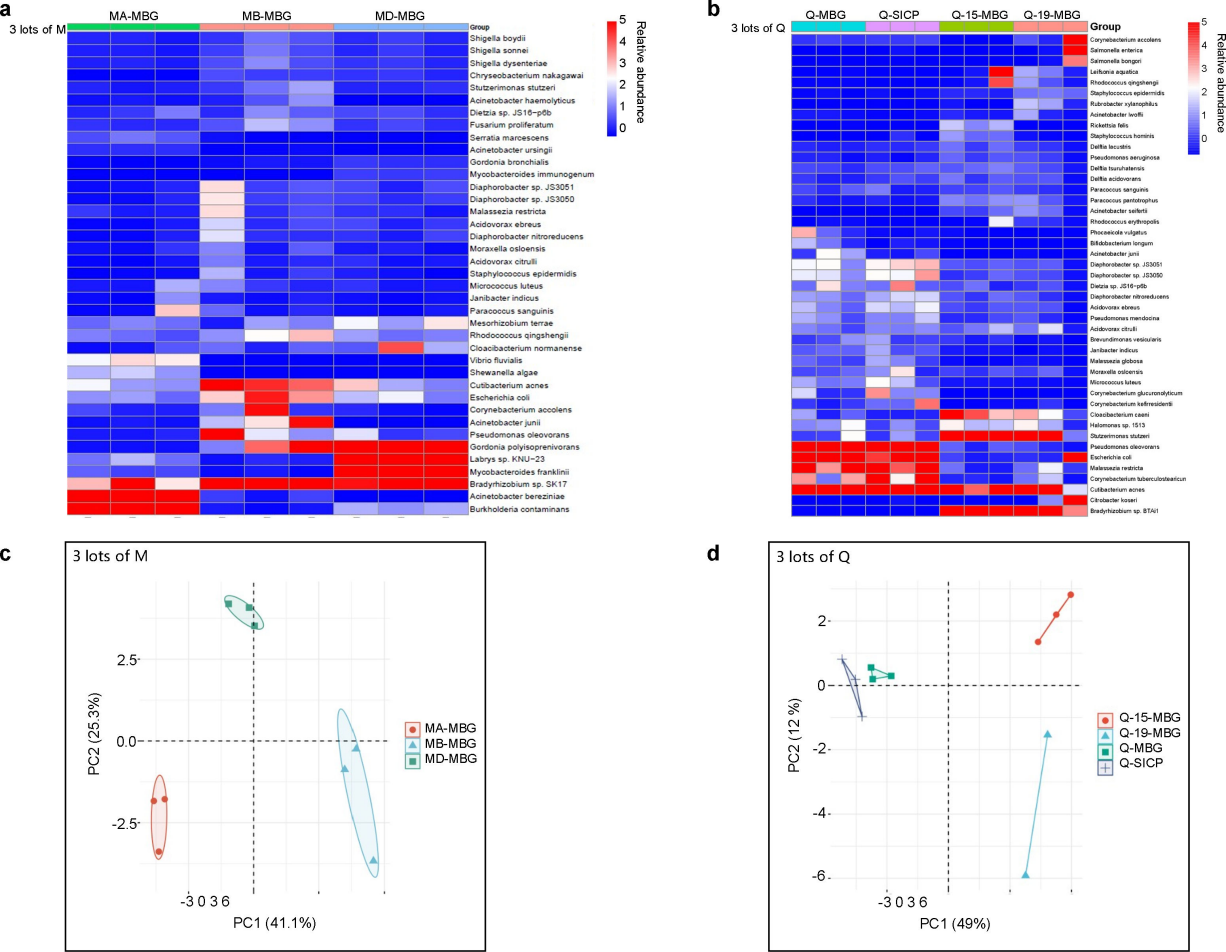
Batch-to-batch variation analysis of extraction kits M and Q. Comparison of background microbiota profiles between different manufacturing lots using molecular-grade (DNA-free) water (MBG) as input. (a) Heatmap showing microbial profiles across three lots of brand M (lots MA, MB, and MD). (b) Heatmap showing microbial profiles across three lots of brand Q (lots Q-MBG, Q-15-MBG, and Q-19-MBG). (c) Principal component analysis (PCA) demonstrating clustering patterns of different lots from brand M. (d) PCA demonstrating clustering patterns of different lots from brand Q.

Analysis revealed that all three lots of M and Q exhibited distinct background microbiota profiles and clustering patterns, suggesting lot-specific backgrounds (Fig. 4). This finding indicates that manufacturers of mNGS extraction kits should profile and provide background microbiota data for each lot. Additionally, manufacturers should identify commonly detected pathogens and ensure that important pathogens would not be masked by high background noise. Laboratories should compare manufacturer-provided background data with their own negative controls to identify laboratory-specific contaminants.

### Comparison between 30 SICP controls done at CMUH

**Fig 5:**
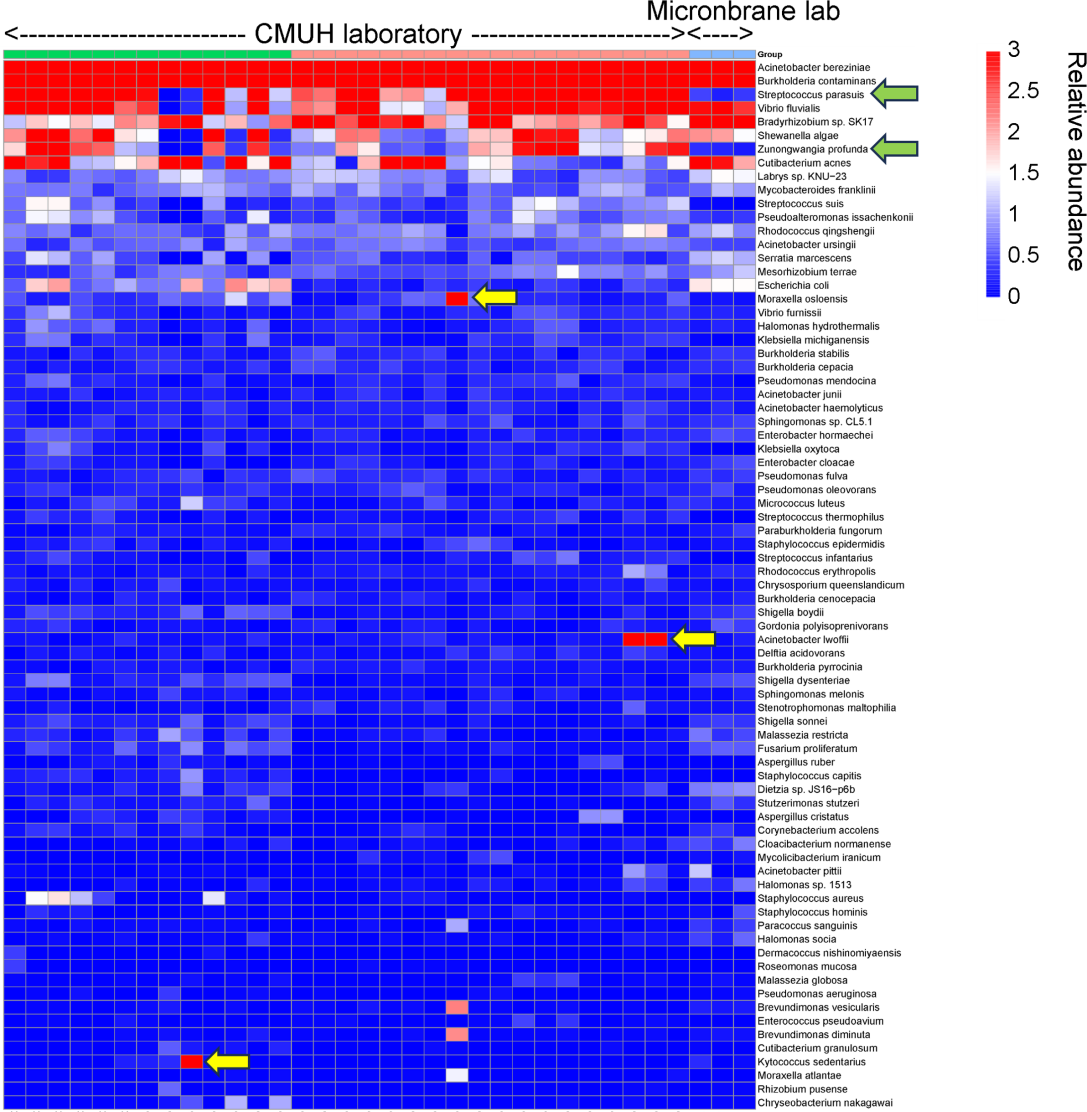
Site-specific contamination analysis between different testing locations. Comparison of background microbiota profiles from ZymoBIOMICS Spike-in Control D6320 (SICP) samples processed at two different locations (Micronbrane laboratory and CMUH laboratory). All samples used the same lot of brand M extraction kit. Green arrows indicate contaminants consistently detected and specific to the CMUH laboratory site (*Streptococcus parasuis* and *Zunongwangia profunda*). Yellow arrows indicate sporadic environmental contaminants unique to the CMUH laboratory (*Moraxella osloensis*, *Kytococcus sedentarius*, and *Acinetobacter lwoffii*).

To investigate run-to-run background noise profiles, we analyzed sequence data from 30 SICP controls performed at CMUH and compared them with SICP data generated at the manufacturer’s (Micronbrane Medical, Taiwan) site. All samples used the same M brand extraction lot. As expected, many contaminants were consistently detected (Fig. 5). Some contaminants were specific to the study laboratory (CMUH), including *Streptococcus parasuis* and *Zunongwangia profunda*. Additional spurious contaminants likely representing environmental contamination specific to the CMUH laboratory included *Moraxella osloensis*, *Kytococcus sedentarius*, and *Acinetobacter lwoffii*. During routine testing, such spurious contaminants can be identified by comparing the negative control of the run against a database of negative controls from past runs in a similar fashion. The same contaminant could then be filtered from the final results, or reported with a warning remark.

### Does a blood microbiome exist in healthy individuals?

**Fig 6:**
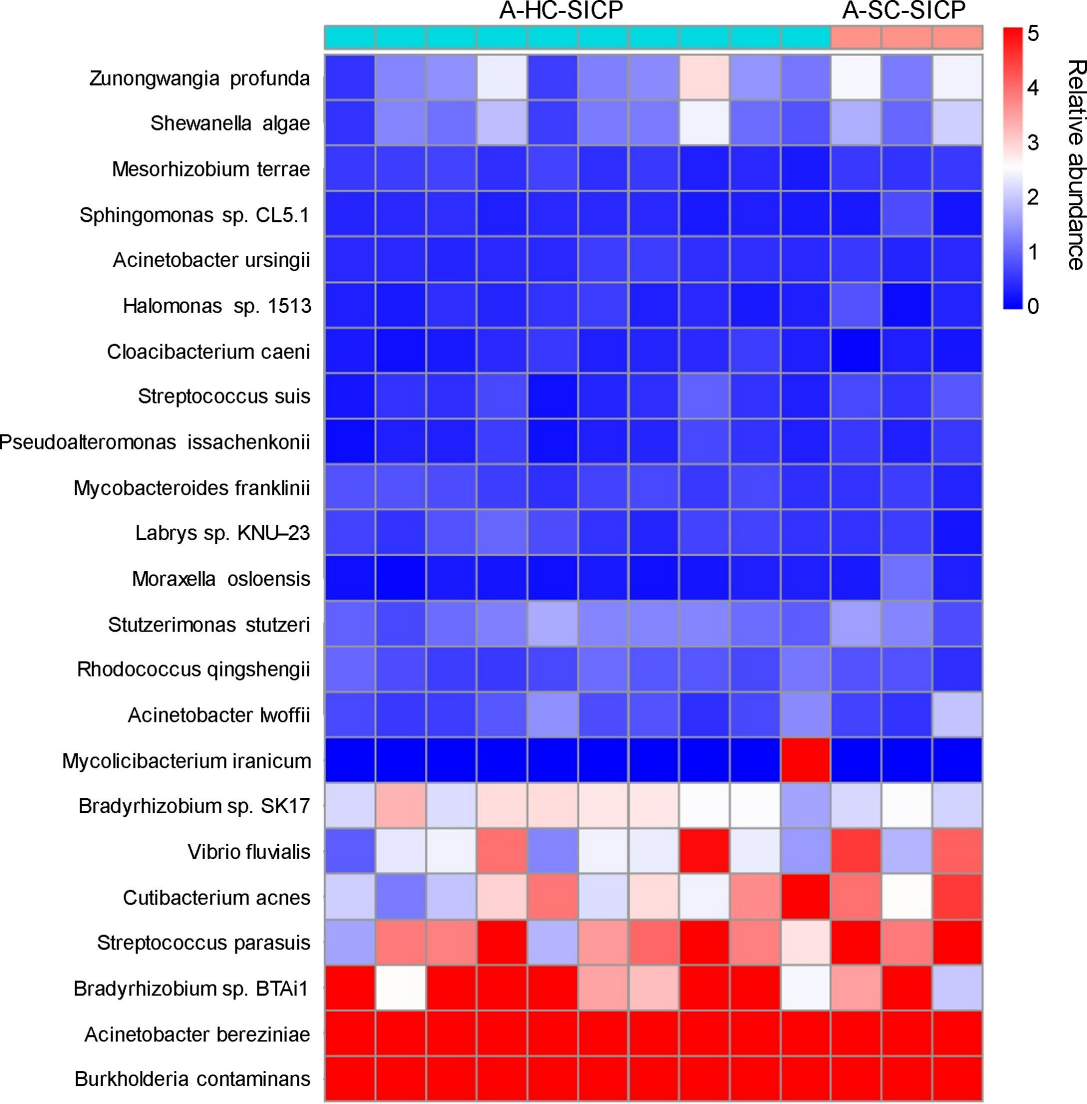
Comparative analysis of microbial profiles from healthy blood samples versus control samples. Comparison of microbial profiles obtained from blood samples of healthy individuals (*n* = 10) and their corresponding ZymoBIOMICS Spike-in Control D6320 (SICP) controls. A heatmap demonstrates the similarity between background microbiota patterns in healthy blood samples and control samples, indicating the absence of a consistent blood microbiome in healthy individuals.

To investigate the existence of a blood microbiome, we compared sequence data from blood samples of 10 healthy controls with their corresponding SICP controls (Fig. 6). Profiles from healthy individuals' blood samples were indistinguishable from SICP samples, suggesting that a consistent “microbiota” cannot be readily detected in healthy blood.

## DISCUSSION

mNGS has emerged as a robust diagnostic tool for infectious diseases, offering a culture-independent and hypothesis-free approach to detect pathogens with known genomic sequences, including bacteria, fungi, viruses, and parasites. However, the results of this study underscore the significant challenges posed by reagent and laboratory contamination in microbiome research. DNA contamination originating from extraction kits, PCR reagents, and laboratory environments remains a critical issue, particularly in low-biomass samples (8, 26). These results demonstrate that reagent-specific and environmental factors jointly impact mNGS workflows (27).

Our findings demonstrate significant differences in background microbiota profiles across reagent brands, as evidenced by the distinct clustering patterns observed among the three tested lots of brands M and Q. These lot-specific variations highlight the critical need for manufacturers to routinely profile and disclose detailed background microbiota data for each batch, thereby ensuring transparency and assisting laboratories in differentiating true microbial signals from contaminant noise. The presence of commonly encountered pathogens in these kits raises concerns about the potential masking of clinically relevant pathogens by high background noise, particularly in low-biomass samples where contaminating signals can predominate. To mitigate these challenges, laboratories should cross-reference manufacturer-provided background profiles with their own negative control results, facilitating the identification of laboratory-specific contaminants. Such practices would not only enhance data reliability but also contribute to more accurate clinical diagnoses and microbiome analyses. These findings underscore the importance of collaboration between manufacturers and end-users to minimize the impact of background noise in mNGS workflows and improve reproducibility across studies. Furthermore, the detection of site-specific contaminants, such as *S. parasuis* and *Z. profunda*, underscores the significant role of laboratory environments in contributing to background noise (Fig. 5). By recognizing unexpected microbial signals as potential contaminants rather than novel discoveries, researchers can improve the accuracy of data interpretation and reduce the risk of erroneous conclusions in microbiome studies (28).

Analysis of blood samples from healthy individuals revealed no consistent blood microbiome, as microbial profiles were indistinguishable from negative controls, including SICP samples. This finding strongly suggests that microbial signals detected in healthy blood are predominantly derived from environmental contamination during sample collection or processing, rather than representing a true endogenous microbiome (29–31). These results align with prior studies indicating that healthy blood lacks a stable or reproducible microbial community and is instead characterized by sporadic and transient contamination events. Indeed, healthy blood is a material that is passed through the same handling pipeline from collection to extraction to sequencing. As such, it makes a much better negative control against multiple sources of contamination in the workflow. It also contains equivalent quantities of non-target/carrier DNA, which is essential for co-precipitation of small quantities of target DNA (32). However, it is operationally difficult to obtain such a control, especially for it to be collected alongside the patient sample. As such, if the inclusion of healthy blood samples as negative controls in routine mNGS testing may be unattainable, extraction blanks may instead serve as the standard negative control for pathogen detection. Bioinformatics tools can further enhance the reliability of analyses by incorporating fold-change calculations relative to these blanks, effectively accounting for reagent-derived contaminants (31, 33). This approach would reduce the risk of misinterpreting contaminating signals as evidence of a blood microbiome, ensuring more accurate and reliable data interpretation in clinical and research settings.

### Conclusion

Our findings underscore the critical need to address reagent and environmental contamination in mNGS, particularly in low-biomass samples where contaminating signals can dominate. By leveraging novel bioinformatics tools and incorporating robust background profiling into clinical workflows, the impact of reagent-derived contaminants can be mitigated, reducing diagnostic errors and improving data reliability. Furthermore, the use of extraction blanks as standard negative controls, rather than healthy blood samples, is essential for ensuring accurate pathogen detection and avoiding misinterpretation of contaminant signals. These strategies are crucial for enhancing the accuracy and reproducibility of mNGS, particularly in settings where the detection of low-abundance microbes is critical for clinical diagnostics and microbiome studies.

## Data Availability

The metagenomic next-generation sequencing (mNGS) data generated in this study are deposited in the NCBI Sequence Read Archive (SRA) under the BioProject accession number PRJNA1190189.

## SUPPLEMENTAL MATERIAL

Table S1 spectrum.03119-24-s0001.docx Comparison of metagenomic sequencing output across different extraction kits M, Q, R, and Z (results from ZymoBIOMICS Spike-in Control D6320 [SICP] samples).

Table S2 spectrum.03119-24-s0002.docx Comparison of metagenomic sequencing output across different extraction kits M, Q, R, and Z (results using molecular-grade [DNA-free] water [MBG] samples).

ASM does not own the copyrights to Supplemental Material that may be linked to, or accessed through, an article. The authors have granted ASM a non-exclusive, world-wide license to publish the Supplemental Material files. Please contact the corresponding author directly for reuse.
