# Supplementary material for: Deciphering the impact of contaminating microbiota in DNA extraction reagents on metagenomic next-generation sequencing workflows
Source: Microbiol Spectr. 2025 Aug 20;13(10):e03119-24. doi: 10.1128/spectrum.03119-24 (PMC12502690; doi:10.1128/spectrum.03119-24)
Supplement: Table S1 — Comparison of metagenomic sequencing output across different extraction kits M, Q, R, and Z (results from ZymoBIOMICS Spike-in Control D6320 [SICP] samples). [file spectrum.03119-24-s0001.docx]

**Supplementary Table S1.** Comparison of metagenomic sequencing output across different extraction kits M, Q, R, and Z. Results from ZymoBIOMICS Spike-in Control D6320 (SICP) samples are shown.

| **Sample** | **Raw reads** | **QC reads** | **QC reads (%)** | **A_halo** | **A_halo (%)** | **I_halo** | **I_halo (%)** | **Sample reads** | **Human reads** | **Human reads (%)** | **Microbial reads** | **Unclassified reads** | **Unclassified reads (%)** |
| --- | --- | --- | --- | --- | --- | --- | --- | --- | --- | --- | --- | --- | --- |
| MEK-01-SICP1 | 803921 | 739132 | 91.94 | 214274 | 28.99 | 421735 | 57.06 | 103123 | 63060 | 61.15 | 24155 | 15908 | 15.43 |
| MEK-01-SICP2 | 490764 | 444877 | 90.65 | 132416 | 29.76 | 252427 | 56.74 | 60034 | 37459 | 62.4 | 14771 | 7804 | 13 |
| MEK-01-SICP3 | 430408 | 385271 | 89.51 | 114570 | 29.74 | 231616 | 60.12 | 39085 | 7862 | 20.12 | 17268 | 13955 | 35.7 |
| Q-SICP1 | 302050 | 272205 | 90.12 | 26390 | 9.69 | 5069 | 1.86 | 240746 | 92508 | 38.43 | 66922 | 81316 | 33.78 |
| Q-SICP2 | 97140 | 85492 | 88.01 | 13940 | 16.31 | 1656 | 1.94 | 69896 | 38932 | 55.7 | 12455 | 18509 | 26.48 |
| Q-SICP3 | 268218 | 235930 | 87.96 | 10493 | 4.45 | 1713 | 0.73 | 223724 | 102882 | 45.99 | 48892 | 71950 | 32.16 |
| R-SICP1 | 317276 | 290162 | 91.45 | 76728 | 26.44 | 52265 | 18.01 | 161169 | 61191 | 37.97 | 21878 | 78100 | 48.46 |
| R-SICP2 | 167799 | 150484 | 89.68 | 62151 | 41.3 | 49899 | 33.16 | 38434 | 7635 | 19.87 | 6475 | 24324 | 63.29 |
| R-SICP3 | 354224 | 325333 | 91.84 | 146060 | 44.9 | 88008 | 27.05 | 91265 | 7652 | 8.38 | 14869 | 68744 | 75.32 |
| Z-SICP1 | 795783 | 726788 | 91.33 | 178398 | 24.55 | 458983 | 63.15 | 89407 | 33787 | 37.79 | 9418 | 46202 | 51.68 |
| Z-SICP2 | 561872 | 509735 | 90.72 | 123336 | 24.2 | 303346 | 59.51 | 83053 | 16117 | 19.41 | 13214 | 53722 | 64.68 |
| Z-SICP3 | 607546 | 511479 | 84.19 | 109711 | 21.45 | 284488 | 55.62 | 117280 | 41507 | 35.39 | 11700 | 64073 | 54.63 |

**A_halo: *Allobacillus halotolerans***

**I_halo: *Imtechella halotolerans***
