# Supplementary material for: Deciphering the impact of contaminating microbiota in DNA extraction reagents on metagenomic next-generation sequencing workflows
Source: Microbiol Spectr. 2025 Aug 20;13(10):e03119-24. doi: 10.1128/spectrum.03119-24 (PMC12502690; doi:10.1128/spectrum.03119-24)
Supplement: Table S2 — Comparison of metagenomic sequencing output across different extraction kits M, Q, R, and Z (results using molecular-grade [DNA-free] water [MBG] samples). [file spectrum.03119-24-s0002.docx]

**Supplementary Table S2.** Comparison of metagenomic sequencing output across different extraction kits M, Q, R, and Z. Results using molecular-grade (DNA-free) water (MBG) samples are shown. 3 lots of MEK01 and Q were tested.

| **Tax** | **Raw reads** | **QC reads** | **QC reads (%)** | **A_halo** | **A_halo (%)** | **I_halo** | **I_halo (%)** | **Sample reads** | **Human reads** | **Human reads (%)** | **Microbial reads** | **Unclassified reads** | **Unclassified reads (%)** |
| --- | --- | --- | --- | --- | --- | --- | --- | --- | --- | --- | --- | --- | --- |
| MEK-01-MBG1 | 478970 | 440567 | 91.98 | 317 | 0.07 | 160 | 0.04 | 440090 | 202936 | 46.11 | 139277 | 97877 | 22.24 |
| MEK-01-MBG2 | 141992 | 132476 | 93.3 | 182 | 0.14 | 204 | 0.15 | 132090 | 39411 | 29.84 | 64842 | 27837 | 21.07 |
| MEK-01-MBG3 | 166584 | 152739 | 91.69 | 239 | 0.16 | 99 | 0.06 | 152401 | 76979 | 50.51 | 44383 | 31039 | 20.37 |
| MEK-01-MBG1-1109211A | 488234 | 425267 | 87.1 | 1751 | 0.41 | 2494 | 0.59 | 421022 | 315997 | 75.05 | 42265 | 62760 | 14.91 |
| MEK-01-MBG2-1109211A | 102131 | 93694 | 91.74 | 210 | 0.22 | 190 | 0.2 | 93294 | 33500 | 35.91 | 32292 | 27502 | 29.48 |
| MEK-01-MBG3-1109211A | 127223 | 116062 | 91.23 | 871 | 0.75 | 882 | 0.76 | 114309 | 60863 | 53.24 | 28373 | 25073 | 21.93 |
| MEK-01-MBG1-M212161A | 122347 | 104589 | 85.49 | 89 | 0.09 | 103 | 0.1 | 104397 | 63606 | 60.93 | 13159 | 27632 | 26.47 |
| MEK-01-MBG2-M212161A | 515580 | 453475 | 87.95 | 277 | 0.06 | 304 | 0.07 | 452894 | 382911 | 84.55 | 28797 | 41186 | 9.09 |
| MEK-01-MBG3-M212161A | 128589 | 117625 | 91.47 | 270 | 0.23 | 199 | 0.17 | 117156 | 38570 | 32.92 | 29454 | 49132 | 41.94 |
| MEK-01-MBG1-MO10191A | 217335 | 196864 | 90.58 | 2277 | 1.16 | 3569 | 1.81 | 191018 | 91234 | 47.76 | 49842 | 49942 | 26.15 |
| MEK-01-MBG2-MO10191A | 136876 | 123714 | 90.38 | 652 | 0.53 | 598 | 0.48 | 122464 | 34563 | 28.22 | 49302 | 38599 | 31.52 |
| MEK-01-MBG3-MO10191A | 274536 | 255032 | 92.9 | 4156 | 1.63 | 6183 | 2.42 | 244693 | 38108 | 15.57 | 114157 | 92428 | 37.77 |
| Q-MBG1 | 170721 | 143422 | 84.01 | 169 | 0.12 | 164 | 0.11 | 143089 | 51943 | 36.3 | 34592 | 56554 | 39.52 |
| Q-MBG2 | 94798 | 77643 | 81.9 | 97 | 0.12 | 40 | 0.05 | 77506 | 26243 | 33.86 | 16498 | 34765 | 44.85 |
| Q-MBG3 | 84102 | 67089 | 79.77 | 106 | 0.16 | 109 | 0.16 | 66874 | 24443 | 36.55 | 14855 | 27576 | 41.24 |
| Q-15-MBG1 | 51663 | 45168 | 87.43 | 25 | 0.06 | 46 | 0.1 | 45097 | 23955 | 53.12 | 7984 | 13158 | 29.18 |
| Q-15-MBG2 | 121728 | 108754 | 89.34 | 85 | 0.08 | 38 | 0.03 | 108631 | 54046 | 49.75 | 25816 | 28769 | 26.48 |
| Q-15-MBG3 | 93016 | 80517 | 86.56 | 104 | 0.13 | 38 | 0.05 | 80375 | 36959 | 45.98 | 15927 | 27489 | 34.2 |
| Q-19-MBG1 | 5466805 | 5220695 | 95.5 | 68 | 0 | 37 | 0 | 5220590 | 5189501 | 99.4 | 15719 | 15370 | 0.29 |
| Q-19-MBG2 | 12299832 | 11711612 | 95.22 | 50 | 0 | 21 | 0 | 11711541 | 11685347 | 99.78 | 12248 | 13946 | 0.12 |
| Q-19-MBG3 | 7924678 | 7551622 | 95.29 | 83 | 0 | 41 | 0 | 7551498 | 7515708 | 99.53 | 26971 | 8819 | 0.12 |
| R-MBG1 | 97638 | 91036 | 93.24 | 45 | 0.05 | 50 | 0.05 | 90941 | 16316 | 17.94 | 16659 | 57966 | 63.74 |
| R-MBG2 | 46892 | 42407 | 90.44 | 47 | 0.11 | 26 | 0.06 | 42334 | 7344 | 17.35 | 6994 | 27996 | 66.13 |
| R-MBG3 | 59277 | 54074 | 91.22 | 59 | 0.11 | 18 | 0.03 | 53997 | 12620 | 23.37 | 10519 | 30858 | 57.15 |
| Z-MBG1 | 658320 | 596795 | 90.65 | 89 | 0.01 | 53 | 0.01 | 596653 | 503160 | 84.33 | 16405 | 77088 | 12.92 |
| Z-MBG2 | 281612 | 258410 | 91.76 | 112 | 0.04 | 94 | 0.04 | 258204 | 160610 | 62.2 | 22002 | 75592 | 29.28 |
| Z-MBG3 | 538443 | 475733 | 88.35 | 149 | 0.03 | 40 | 0.01 | 475544 | 324824 | 68.31 | 31652 | 119068 | 25.04 |
